# The Education of Mr. T

**DOI:** 10.1371/journal.pbio.0040117

**Published:** 2006-03-07

**Authors:** Caitlin Sedwick

T “helper” cells (which express the surface marker CD4) and “killer” T cells (which express CD8 markers) are each critical for detecting and neutralizing microbial invaders and protecting the body from disease. Both types of T cells recognize foreign invaders through surface expression of a T cell receptor (TCR) that is unique to each T cell. When an infected cell expresses protein fragments (peptides) derived from a pathogen on its surface, it raises a red flag for the TCR that recognizes the peptide.

Before CD4 T helper cells or CD8 killer T cells can be unleashed on invading armies of microbes, they must first learn how to detect appropriate targets for their activities. This education process takes place in the thymus (thus, the “T” in their name), where T cells originate. Immature cells that will eventually become T cells come to the thymus from the bone marrow. Once they arrive in the thymus, immature T cells (now called “thymocytes”) undergo specific maturation steps that result in the simultaneous surface expression of both CD8 and CD4 proteins.

Later, they will choose to express only one of these determinants, but these “double-positive” thymocytes must first pass two sequential life-and-death tests. First, they undergo positive selection to make sure they have a functional TCR. Then they undergo negative selection to ensure that their TCR does not strongly recognize determinants derived from body proteins (self). If a prospective T cell expresses a defunct TCR on its surface, it will fail the positive selection test and undergo “death by neglect.” Then, if it expresses a TCR that responds too enthusiastically, it will fail the negative selection test and will also die. When T cell education goes awry, an individual faces dire consequences, including severe immune deficiency or autoimmune disease.

In both positive and negative selection, T cell death is accomplished by a process called apoptosis (cellular suicide), in which the Bcl-2 family of proteins plays a much-celebrated role: some Bcl-2 family members (including Bcl-2 and Bcl-xL) protect cells against apoptosis, while others (such as Bax, Bak, and Bid) promote it. Scientists still struggle to understand how the activity of Bcl-2 proteins is regulated inside cells.

In an effort to identify other proteins involved in the education of T cells, Takeshi Nitta, Yousuke Takahama, and their colleagues undertook a screen for genes whose expression changes in T cells during positive selection. The screen identified the immune-associated nucleotide-binding (IAN; also known as GIMAP) family of proteins, which are all expressed in immune tissues, as players in this process. In particular, the researchers identified *IAN1*, *IAN4*, and *IAN5* as participating in the process of T cell education, through an interaction with various Bcl-2 family members.

Nitta and his colleagues first characterized the expression pattern of the IAN family generally: in the mouse, there are eight IAN family proteins (IAN1–7 and IAN9), whose coding genes are all packed together on Chromosome 6. As expected, they found the genes of these proteins expressed predominantly in immune tissues (thymus, spleen, lymph nodes, and bone marrow) and also in the lung. When they looked for expression of these proteins in T cells, they found that expression of *IAN1*, *IAN4*, and *IAN5* increased in thymocytes during the process of positive selection.

To further probe the functions of these proteins, the authors engineered thymocytes to prematurely overexpress either *IAN1*, *IAN4*, or *IAN5*. Under these conditions, cells overexpressing *IAN1* died prematurely (at the CD4, CD8 double-positive stage), while those expressing *IAN4* or *IAN5* were not affected. Conversely, when Nitta and his collaborators prevented thymocytes from expressing these proteins, they found that loss of IAN4 expression blocked positive selection, while loss of IAN5 prevented thymocytes from reaching the double-positive stage; loss of IAN1 expression had no effect. Subsequently, the authors found that IAN4 and IAN5 each associate with Bcl-2 proteins (of both the pro- and anti-apoptotic flavors), while IAN1 was found to associate specifically with Bax; the intracellular location of the individual IAN proteins also co-localized with their respective Bcl-2 binding partners.

Because the expression of these three IAN proteins is carefully timed during T cell development—and because they interact with Bcl-2 family members and appear to regulate T cell survival—further investigation is warranted to determine the precise contribution of IAN proteins to the T cell developmental and survival process. There are also several questions remaining to be examined, including the structural basis of the IAN: Bcl-2 family protein association. The identification of IAN proteins' involvement in T cell development opens a rich trove of potential investigations.

## 

**Figure:**
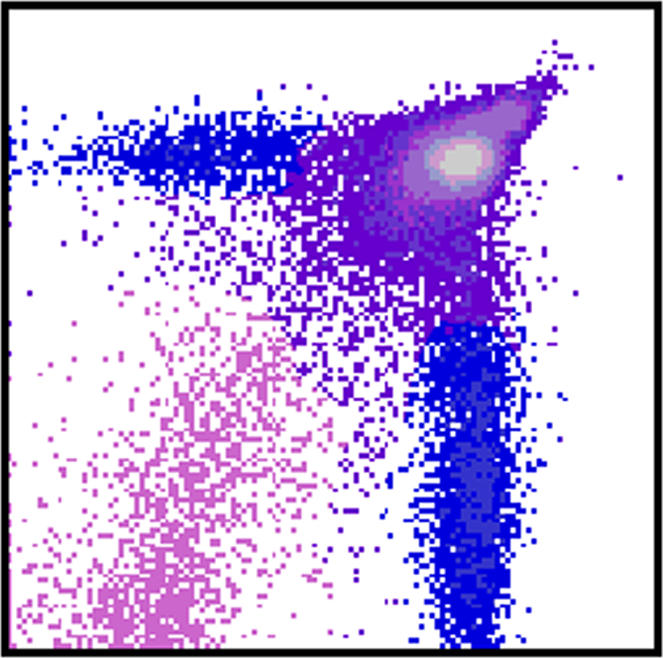
A representative flow cytometry profile of CD4 and CD8 expression in adult mouse thymocytes. Researchers can use such data to track the progression of thymocytes through different developmental stages.

